# Correction: A bicycle can be balanced by stochastic optimal feedback control but only with accurate speed estimates

**DOI:** 10.1371/journal.pone.0317779

**Published:** 2025-01-15

**Authors:** The PLOS ONE Staff

In the Stochastic OFC deals with noise in an optimal way subsection of Results, the equation 14 is formatted incorrectly. Please view the correct equation 14 here.


$$J=\underset{T\to \infty }{\text{lim}}\frac{1}{T}E\left(\overset{T}{\underset{0}{\int }}\left[x{\left(t\right)}^{\prime }Qx\left(t\right)+u{\left(t\right)}^{\prime }Ru\left(t\right)\right]dt\right)$$

The publisher apologizes for the error.
