# Supplementary material for: A bicycle can be balanced by stochastic optimal feedback control but only with accurate speed estimates
Source: PLoS One. 2023 Feb 27;18(2):e0278961. doi: 10.1371/journal.pone.0278961 (PMC9970107; doi:10.1371/journal.pone.0278961)
Supplement: Attachment — Submitted filename: BalanceBicycleOFCReply2PlosCompBiolReviews_20220818.docx [file pone.0278961.s002.docx]

# A bicycle can be balanced by stochastic optimal feedback control but only with accurate speed estimates

## Point-by-point reply to the reviewers of PLOS Computational Biology

The reviewers’ comments are written in roman typeface and my replies in italic.

## Reviewer #1

The paper presents a detailed modelling study on the ability of stochastic optimal control model (SOC) to balance a bicycle. Attention is given to the robustness of the model against variation or error in model parameters including noise covariance matrices and velocity. The quality of the text and of the results is very high and the material is presented in very clear and readable way. I however have reservation about the contents of the paper for two main reasons. The first is that the title is misleading, it is not truly a bicycle but a simplified version of the bicycle that can be balanced. The author presents a fair discussion about the simplifying assumptions but the reader is left wondering whether any of the result still holds should a more complex model be considered. This is however a fair limitation that impacts most modelling studies.

*In the revised version of the manuscript, I have now addressed all research questions using two instead of a single bicycle model. The new bicycle model, the benchmark double pendulum (BDP), has a front frame with self-stabilizing forces (gyroscopic forces plus the forces that depend on geometric trail and wheel flop). The BDP is a linear model, whereas the original bicycle model, the steered double pendulum (SDP), is nonlinear. Although the stabilization of the BDP is much more robust to noise, the pattern in the simulation results is highly similar for the two bicycle models (stabilization under realistic conditions, robustness to inaccurate sensorimotor noise characteristics, and extreme sensitivity to inaccurate speed estimates). This extension of the manuscript thus demonstrates the generality of the stabilizing performance of stochastic OFC across linear and nonlinear bicycle models with and without self-stabilizing forces, but also highlights the pervasive nature of the sensitivity to inaccurate speed estimates.*

The second problem is that, as a consequence, the simplified model is more of a thoughtful and careful exercise, which is expected to work, and which does not provide much conceptual advance or novelty. At the end of the paper I felt that the modelling work was squarely in line with standard SOC models, and that the insight that is provided under all assumptions made was that balancing a bike required good estimates of velocity. It is likely sound but perhaps falls a bit short of the scientific advance that is expected for the journal. It does not take anything away from the quality of the manuscript. If there is a more important or fundamental contribution that I missed it should be spelled out in more detail.

*I agree that the scientific advance provided by my results could have been highlighted better. In fact, the extreme dependence of balancing performance on accurate speed estimates is a very important observation. Specifically, if the dynamics of a mechanical system strongly depends on a parameter (here, speed), then the computational system must be able to switch between different internal models that approximate the mechanical system for different values of this parameter. Crucially, this switch must be controlled by a reliable index of this parameter, and the most likely source of information is sensory input. However, for movement speed, neither visual (optical flow) nor proprioceptive information (pedaling cadence) are obvious candidates. I have added the following paragraph to the Discussion section:*

“The first useful extension immediately follows from the third simulation study: if the model must apply to a wide range of speeds, a mechanism must be added for accurate speed estimation and selection of the appropriate system matrix. For motor control in general, this idea also has been proposed by Scott (1), but focusing only on the feedback (LQR) gain matrix (which depends on the system matrix). The question now is “What is the speed-related information based on which the appropriate system matrix is selected?”. Because of its large Weber fraction [2], it is doubtful that optic flow is the only source of information for speed estimation. This is supported by the fact that many experienced cyclists can ride on stationary bicycle rollers. Moreover, they can do so while shifting gears, and this is inconsistent with a simple readout of proprioceptive information (pedaling cadence) as a substitute for the missing optic flow. I conclude that none of the known sources of sensory information is a plausible candidate for selecting the appropriate system matrix.”

I also have a number of major concerns listed below:

-	I was confused about the learning that is mentioned throughout the paper. It is said multiple times that model parameters and noise covariance matrices are learned but if I am not mistaken the learning procedure or learning rule is not part of the study. I would avoid any emphasis on learning if this is not an explicit component of the paper, simply the author assumes that these parameters are known, it is clear that they were learned through practice.

*As much as possible, I have replaced “learned” by “known”. However, there are a few paragraphs for which it is helpful to have the context of a biological organism that acquires an internal model at a time scale that almost always corresponds to learning. In those paragraphs, I kept the adjective “learned”.*

-	I found that there was a clear lack of basic simulations results to give more intuition to the reader. The paper jumps quite directly to maximum angles as a function of parameters, which is quite difficult to relate to system behaviour. It is difficult to understand how these maximum angles relate to actual trajectories. It seems necessary to simply plot the main or relevant raw state variables as a function of time to visualise stabilisation properties and link the system trajectories to the figures about robustness.

*I understand this criticism, and I have now presented my simulation results in terms of two variables that a reader can visualize easily: the curvature of the turns and the lean angle of the combined CoG. To visualize the actual trajectories, readers can use the Matlab code that is shared in the Supporting Information, which includes a visualization routine that shows simulated trajectories as a movie. I have shared a few movies on a Youtube channel (see,* [*https://youtu.be/hbJZC9xhnnk*](https://youtu.be/hbJZC9xhnnk)*). To get a feel for the simulated behavior, the dynamical aspect of the simulation, as shown in a movie at the appropriate speed, is essential.*

-	The results about the robustness against errors in noise parameters may be erroneous. First, saying that it is robust against learned motor noise covariance that are too small. This may simply result from the use of additive noise model; in this case the expected cost is minimized and the covariance only offsets the cost-to-go but leaves average trajectories unchanged. In practice however larger variability on a bike is likely not tenable and I find it difficult to believe that this result translates into a genuine property of biological control. Second, it is said that the model is robust against sensor noise covariances that are too large. Again, this observation is artificial: larger sensory covariance matrices only slow down any change in the estimate by lowering the Kalman gain. It is well known that in the settings of additive noise, the stability of the closed loop dynamics is not threatened by the dynamics of the estimate, so larger covariances will only make the

estimate a little sloppier without impact on the dynamics. As before this probably does not translate to a genuine property of balance control, since a sloppier estimate will reduce the system robustness to external disturbances (because it will be slower to respond), like when one rides on a bumpy road. I admit I have doubts about the validity of the interpretation of the results on robustness against noise parameters in the context of human motor control. There is also a rich literature on robustness against noise parameters in control theory that is not represented here (see e.g. Nishimura, 1966, 10.1109/tac.1966.1098321 and citing references).

This type of robustness (to inaccuracies in motor noise covariance matrices that are too small and sensor noise covariance matrices that are too large) is specific to the SDP. The revised manuscript also describes the simulations for the BDP, and for this bicycle model the robustness is uniform across the noise levels. Thus, the robustness results for the SDP are not a genuine property of biological control. It was never my intention to draw this conclusion. Instead, I want to argue that the extreme sensitivity to misestimation of the bicycle speed is not due to a general lack of robustness of the model for balance control. This is also expressed clearly in the Discussion section:

“For the BDP, this robustness is uniform across the noise levels, but for the SDP, stabilization performance is only robust to learned motor noise covariances that are too small and learned sensor noise covariances that are too large. The third simulation study shows that, compared to the robustness to inaccuracies in the learned noise covariance matrices, the stabilization is much less robust to inaccuracies in the system matrix that result from misestimation of the bicycle speed.”

Specific comments:

-Line 56: this condition is under the hypotheses of the model, so the implication is not established.

*I removed this sentence.*

-Lines 78-80: denoting inverse models by a feedback control law is inaccurate. They are different concepts.

*Different authors in the field think differently about this conceptual issue [see, e.g., 3]. However, I removed the statement that equated the inverse model and the feedback control law.*

- I am not expert in the physics of cycling but I was under the impression that the stabilising effect of speed was pushed aside. Clearly with speed there is no need to stabilise as much as with very low velocity. Was it considered? I did not follow well the paragraph at line 267.

*This is a difficult point to address in the present manuscript because it pertains to*

*a subtle issue in bicycle mechanics: after some speed, most bicycles are no longer self-stable, but their dynamics is dominated by a mode (the caster mode) that is very controllable. To bring across this point in a self-contained manuscript requires a formal distinction between self-stability (which depends on the eigenvalues of the system matrix) and controllability (which depends on the controllability matrix). This would require too much text being spent on a topic that is not central to the manuscript.*

-line 334: It is not true that there are two versions of optimal control. This is a recurrent misconception in the field to categorise optimal control with or without trajectory tracking but in fact tracking tasks can be expressed in OFC framework.

*I agree, and I have rewritten this paragraph such that this misunderstanding cannot occur anymore:*

“OFC involves that the control actions are chosen such that this cost functional is minimized. OFC is sometimes proposed as an alternative for feedforward trajectory planning [4], but this incorrectly suggests that OFC and trajectory planning cannot be combined. In this paper, I will present a model for the specific task of balance control, as when cycling blindfolded on an empty parking lot. For this specific task, I can ignore all aspects of cycling that involve trajectory planning, such as steering a bicycle over an indicated path or an obstacle course.”

-436: that the model with wider domain of parameters is more likely to be used in the CNS is not supported empirically or theoretically.

*I agree. I have toned down the paragraph as follows:*

“The larger the range of parameter and state values for which the internal model can stabilize the nonlinear system, the more robust the control. Because the CNS must learn the internal model from experience with the mechanical system, which may be a slow process, the imperfect internal must be robust to some inaccuracies in the internal model.”

-470: Pls add information about the linearization at this stage (around an equilibrium? A trajectory?)

*I did so by changing two sentences in this paragraph:*

“In its application to balancing a bicycle, this mechanical system corresponds to the rider’s body plus his bicycle, and the linearization is around the equilibrium point (unstable fixed point). In other applications, the mechanical system may also involve objects in the environment that are sensed from a distance using vision and/or audition, and the linearization may be around a trajectory.”

-In general, the paper reads a bit as a lengthy course on modelling the task of balancing a bicycle, perhaps for the journal’s audience it is fine to be more concise. That linearized dynamics is used to derive an LQG controller is fairly standard.

*From the perspective of motor control, the detail on modelling the task may be too much, but from the perspective of bicycle dynamics it probably is not. Also, no detail is provided on the linearization of the SDP, because the Jacobian was obtained by Matlab’s Symbolic Toolbox and not as a set of equations.*

-608: What does it mean that the matrices are worse?

*I agree, this is unclear. I have removed this sentence.*

-883: word missing?

*I did not want to use the word “system” twice, but this has resulted in a sentence that is difficult to parse. In the revised manuscript repeat the word “system”.*

-885: It should be clarified that what follows is a consequence of the modelling assumptions, cation is warranted before making them principles of sensorimotor control.

I think this should be obvious from the sentence, which states “… the stabilization performance **of the model** …”.

## Reviewer #2

In the submitted manuscript, the author describes his work developing a controller for a bicycle and testing the controller in computer simulations. The author developed the dynamical model of the system (a simplified bicycle and rider), which is similar to a double inverted pendulum on a cart. The author finds that the controller produces control signals that are consistent with human capability and can stabilize a bicycle. However, the model is very sensitive to speed.

I appreciate the author’s efforts in completing this work and the different perspectives on bicycle control. The author appears to have significant expertise in motor control and internal models, but not expertise in engineering mechanics. Due to flaws in the modeling and disregard for existing knowledge on the dynamics of singletrack vehicles (bicycles and motorcycles), I do not feel this manuscript in its current form is a meaningful contribution to the literature.

My major concerns are as follows:

1. The majority of the introduction includes no citations to support the author’s statements. The definition of balance for walking, for example, is well-defined in the literature.

2. There has been significant, serious, and quality research on bicycle dynamics in the past 20 years. While the author does identify a subset of the published works, there also exists significant work examining the human control of the bicycle – most work in this area has been led by Dr. Arend Schwab or Dr. Jason Moore. The current study is not a rigorous scientific study, as it largely ignores the existing knowledge and best approaches for evaluating control models.

3. The author developed his own model of the bicycle rather than using the validated equations of motion for a bicycle. The bicycle the author uses can easily be obtained using the bicycle model of Meijaard et al. 2007 by simply choosing parameter values. The author spends way too much of the paper talking about the development of his own model, which would be unnecessary if he simply utilized the established linearized equations of motion for a bicycle.

4. The model that the author uses contains major errors/flaws. Steering torque and lean torque do not have any damping or stiffness! While the equations of motion for a bicycle do have damping and stiffness-like matrices, these terms are a result of the dynamics of a bicycle. In Meijaard et al. 2007, the authors even state “The damping-like (there is no real damping) matrix C = vC_1 is linear in the forward speed v and captures skew-symmetric gyroscopic torques due to steer and lean rates.” Equation 8 is absolutely incorrect, as is the calculation of a damping ratio and time constant for steering torque. This is not how one models a torque input to a bicycle (or any singletrack vehicle).

5. If the model was correct (which it is not), the evaluation of the controller is based only on whether the author thinks the maximum control responses are reasonable. The values are not based on scientific knowledge about strength or movement speed.

6. Overall this article is far too long and does not recognize or utilize existing knowledge about bicycle control. As a result, the reader must use a lot of time and energy learning about a model that has unclear value for understanding real human balance control.

*Because I disagree with most of these points and because Reviewer #2 revealed his name, I wrote him an email with a few clarifications and some questions. I have pasted this email below.*

*In reply to the reviewer’s main criticism, not making use of the linearized equations of motion of Meijaard, Papadopoulos (5), I extended this model with a third rigid body (the rider’s upper body) and did my three simulation studies also for the resulting linear bicycle model (the BDP). For all three simulation studies, the same conclusion can be drawn for the original nonlinear SDP as for the linear BDP. All three simulation studies also showed that the BDP is much less susceptible to noise than the SDP, and this is probably due to the positive mechanical trail of the BDP front frame. This positive mechanical trail generates caster forces in the front frame which reduce the impact of the noise on the handlebars by aligning the disturbed front wheel with the non-disturbed rear frame [6].*

*My email to Reviewer #2:*

*“You signed your review of the paper that I submitted to Plos Computational Biology. From that, I infer that you don’t mind that I contact you. I also sign my reviews, and this has already resulted in a few interesting discussions. If you don’t want to engage in an exchange of ideas, just tell me, and I will understand.*

*The submitted paper needs improvement on a number of points, and the reviews were very useful in that respect. However, there were a few points in your review that I did not understand, and probably this is due to the fact that I did not explain my objectives well enough.*

1. *Starting with the seminal paper by Todorov & Jordan (2002), in the field of motor control, there is a lot of interest in how the brain can control a nonlinear mechanical system (the body’s limbs and the tools attached to it; e.g. a bicycle) on the basis of a linear approximation of this system. If I would start from the linearized bicycle model of Meijaard et al (2007), then there would be no difference between the system to be approximated and the approximation (as both are linear). Many of my colleagues in motor control would not be very interested in a demonstration of a linear mechanical system being controlled by a linear controller.*
2. *You state that the model in the paper contains major errors/flaws, and you point to Equation 8, which describes the torsional spring-mass-damper system for the steering angle. (Although you did not mention this, your critique probably also applies to Equation 9, which contains damping and stiffness terms for the pelvis.)  You refer to Meijaard et al (2007), whose linearized equations do not contain real damping or stiffness. Your express this critique probably because I did not stress sufficiently that the damping and stiffness are properties of the rider’s body (front arms for the steering angle subsystem, and the pelvis for the roll angle subsystem). I am not 100% happy with the spring-mass-damper systems for steering and the pelvis movements. However, (1) it is the best I could find, (2) I selected realistic parameter values, (3) the results are robust to large changes in these parameter values (order of magnitude; unpublished results), and (4) everyone in the field of motor control is using it. In any case, it is the least one should do if one models a rider that can provide control input to the front- and the rear frame. It is clear that there is no real damping and stiffness in the Meijaard et al (2007) model, but this is a model that contains a passive rider that is rigidly attached to the rear frame.*

*I must admit that I find it very difficult to collaborate with my colleagues in the engineering departments. My real interest is in how the brain builds an internal model about the mechanical properties of the body and the attached tools, and it is not my ambition to contribute on the mechanical properties of the tools (the bicycle) in isolation from the human (the rider). I would be happy to use an extended version of the Meijaard et al (2007) model for the paper (stiffness and damping terms added), if that would result in a more positive review by the engineering community. I still think that successful linear control of a nonlinear system would be more convincing, but the spectacular dependence on an accurate speed estimate can also be made using the Meijaard et al (2007) model. Actually, I did the simulations already a while ago, and I only have to do the writing.*

*I am a bit puzzled by your statement that Schwab & Moore have done important work on human control of the bicycle. I only know about a conference proceedings and a VSD paper, and both left me puzzled:*

1. *Schwab, A. L., De Lange, P. D. L., & Moore, J. K. (2012, October). Rider optimal control identification in bicycling. In Dynamic Systems and Control Conference (Vol. 45318, pp. 201-206). American Society of Mechanical Engineers.*
2. *Schwab, A. L., Meijaard, J. P., & Kooijman, J. D. G. (2012). Lateral dynamics of a bicycle with a passive rider model: stability and controllability. Vehicle system dynamics, 50(8), 1209-1224.*

*The conference proceedings paper describes a PID controller but I could not find any results on its performance. The second paper is really interesting, but I found it extremely difficult to relate it to human balance control. I learned from this paper that riding position dramatically affects stability of the passive rider system because small gravitational force at the handlebars may destroy the bicycle’s self-stability. The second part of the paper is about the active rider system (either steering or lean angle control) and is investigated by characterizing the null space of the controllability matrix (actually, its transpose). This is very interesting from a robotics perspective (stabilizing a riderless bicycle by means of a motor that applies a torque to either the steering axis or the rear frame), but I find it difficult to see the relevance for a human controller, which produces noisy neuromuscular output on the basis of a noisy state estimate. At the end, I decided to not refer to this paper because the idea of a discrete set of velocities at which the bicycle is uncontrollable (which is central to the second part of this paper) will leave all my neuroscience colleagues puzzled. In retrospect, I now see that I should have referred to this paper, and maybe you can give me your view on what is the crucial relevance for the human controller. Probably I am also missing other relevant work by Schwab & Moore, and maybe you can give me a pointer.*

*Maybe you can also give me a pointer to a reference on dynamic balance. Your first major concern states “The majority of the introduction includes no citations to support the author’s statements. The definition of balance for walking, for example, is well-defined in the literature.” For static balance, most papers cite one of papers by Winter (mostly 1995), but I could not find such a reference for dynamic balance. The idea of bringing the area of support under the CoM can be found in several papers, but nobody seems to have claimed it. What would you cite?*

*Unrelated to the paper that I submitted, I should tell you that I have recently completed a bicycle simulator that is strongly inspired by the bicycle rollers setup that you used for your 2016 Plos One paper. I managed to build a setup that is 180 cm wide, which requires a very similar type of balance control as the one on a regular Dutch bike path. The focus is on kinematics and I collect my data using an Optotrak system. There is lot to be said about this, and I would be happy to discuss this with you. When you are in Europe, I would happy to host you in Nijmegen.”*

*In reply to this email, Reviewer #2 forwarded a link to a 2022 paper from Arend Schwab’s group:* [*https://www.tandfonline.com/doi/full/10.1080/00423114.2022.2048865*](https://urldefense.com/v3/__https:/www.tandfonline.com/doi/full/10.1080/00423114.2022.2048865__;!!HJOPV4FYYWzcc1jazlU!_P8V796K9ppanXcAcEExVIzLbIt7zv8ow2DplVYl-G4vzoRrSyU_9-Kslw3jqOAMlg7rHjOOblPVdktBM27nAyRqmUkpr_MI$) *[7]. This paper relevant to the present manuscript, and I cited it in the appropriate paragraph.*

## References

1. 1.	Scott SH. The computational and neural basis of voluntary motor control and planning. Trends in cognitive sciences. 2012;16(11):541-9.
2. 2.	Monen J, Brenner E. Detecting changes in one's own velocity from the optic flow. Perception. 1994;23(6):681-90.
3. 3.	Todorov E, Jordan MI. Optimal feedback control as a theory of motor coordination. Nature neuroscience. 2002;5(11):1226-35.
4. 4.	Todorov E. Optimality principles in sensorimotor control. Nature neuroscience. 2004;7(9):907-15.
5. 5.	Meijaard JP, Papadopoulos JM, Ruina A, Schwab AL, editors. Linearized dynamics equations for the balance and steer of a bicycle: a benchmark and review. Proceedings of the Royal Society of London A: Mathematical, Physical and Engineering Sciences; 2007: The Royal Society.
6. 6.	Cossalter V. Motorcycle dynamics: Lulu. com; 2006.
7. 7.	Dialynas G, Christoforidis C, Happee R, Schwab A. Rider control identification in cycling taking into account steering torque feedback and sensory delays. Vehicle System Dynamics. 2022:1-25.
