# Supplementary material for: A bicycle can be balanced by stochastic optimal feedback control but only with accurate speed estimates
Source: PLoS One. 2023 Feb 27;18(2):e0278961. doi: 10.1371/journal.pone.0278961 (PMC9970107; doi:10.1371/journal.pone.0278961)
Supplement: Attachment — Submitted filename: BalanceBicycleOFCResponseToReviewers_20221111.docx [file pone.0278961.s003.docx]

# A bicycle can be balanced by stochastic optimal feedback control but only with accurate speed estimates

## Point-by-point reply to academic editor and reviewers

The comments are written in roman typeface and my replies in italic.

## Academic editor

1. Please ensure that your manuscript meets PLOS ONE's style requirements, including those for file naming. The PLOS ONE style templates can be found at

[https://journals.plos.org/plosone/s/file?id=wjVg/PLOSOne_formatting_sample_main_body.pdf](https://urldefense.com/v3/__https:/journals.plos.org/plosone/s/file?id=wjVg*PLOSOne_formatting_sample_main_body.pdf__;Lw!!HJOPV4FYYWzcc1jazlU!-TB1gFgg-DDkhWFMhhZYB8x2uelv2Rci6tav-fvkeLy4SwwpANwln7bxF0pMrcR9TabdKXrlwibPiXbQKg0NjA$) and

[https://journals.plos.org/plosone/s/file?id=ba62/PLOSOne_formatting_sample_title_authors_affiliations.pdf](https://urldefense.com/v3/__https:/journals.plos.org/plosone/s/file?id=ba62*PLOSOne_formatting_sample_title_authors_affiliations.pdf__;Lw!!HJOPV4FYYWzcc1jazlU!-TB1gFgg-DDkhWFMhhZYB8x2uelv2Rci6tav-fvkeLy4SwwpANwln7bxF0pMrcR9TabdKXrlwibPiXbdRH3tmw$)

*I made the following changes to the paper’s style: (1) I placed the Methods section after the Introduction, and (2) I changed the formatting of the headings.*

2. We note that the grant information you provided in the ‘Funding Information’ and ‘Financial Disclosure’ sections do not match.

When you resubmit, please ensure that you provide the correct grant numbers for the awards you received for your study in the ‘Funding Information’ section.

*I ticked the box “The author(s) received no specific funding for this work.” and did not add a Financial Disclosure section because this was not asked for. Should I do something more?*

3. In your Data Availability statement, you have not specified where the minimal data set underlying the results described in your manuscript can be found. PLOS defines a study's minimal data set as the underlying data used to reach the conclusions drawn in the manuscript and any additional data required to replicate the reported study findings in their entirety. All PLOS journals require that the minimal data set be made fully available. For more information about our data policy, please see [http://journals.plos.org/plosone/s/data-availability](https://urldefense.com/v3/__http:/journals.plos.org/plosone/s/data-availability__;!!HJOPV4FYYWzcc1jazlU!-TB1gFgg-DDkhWFMhhZYB8x2uelv2Rci6tav-fvkeLy4SwwpANwln7bxF0pMrcR9TabdKXrlwibPiXYCaRLvaw$).

Upon re-submitting your revised manuscript, please upload your study’s minimal underlying data set as either Supporting Information files or to a stable, public repository and include the relevant URLs, DOIs, or accession numbers within your revised cover letter. For a list of acceptable repositories, please see [http://journals.plos.org/plosone/s/data-availability#loc-recommended-repositories](https://urldefense.com/v3/__http:/journals.plos.org/plosone/s/data-availability*loc-recommended-repositories__;Iw!!HJOPV4FYYWzcc1jazlU!-TB1gFgg-DDkhWFMhhZYB8x2uelv2Rci6tav-fvkeLy4SwwpANwln7bxF0pMrcR9TabdKXrlwibPiXYmqRxvWg$). Any potentially identifying patient information must be fully anonymized.

Important: If there are ethical or legal restrictions to sharing your data publicly, please explain these restrictions in detail. Please see our guidelines for more information on what we consider unacceptable restrictions to publicly sharing data: [http://journals.plos.org/plosone/s/data-availability#loc-unacceptable-data-access-restrictions](https://urldefense.com/v3/__http:/journals.plos.org/plosone/s/data-availability*loc-unacceptable-data-access-restrictions__;Iw!!HJOPV4FYYWzcc1jazlU!-TB1gFgg-DDkhWFMhhZYB8x2uelv2Rci6tav-fvkeLy4SwwpANwln7bxF0pMrcR9TabdKXrlwibPiXYKliyl9A$). Note that it is not acceptable for the authors to be the sole named individuals responsible for ensuring data access.

We will update your Data Availability statement to reflect the information you provide in your cover letter.

*I’m not sure what I should do additionally beyond what I did for the initial submission. In my Data Availability statement I have indicated that “All relevant data are within the manuscript and its Supporting Information files.”. This paper uses simulations to investigate whether a computational model is plausible for actual bicycle balance control. The documented computer code (Matlab Live scripts) to run these simulations and to extend on them, are provided in the Supporting Information. No experiment has been conducted to test this model, but we make use of empirical information. In fact, we know from experience what turn radiuses are physically possible, and what lean angles are acceptable for a rider.*

4. Please review your reference list to ensure that it is complete and correct. If you have cited papers that have been retracted, please include the rationale for doing so in the manuscript text, or remove these references and replace them with relevant current references. Any changes to the reference list should be mentioned in the rebuttal letter that accompanies your revised manuscript. If you need to cite a retracted article, indicate the article’s retracted status in the References list and also include a citation and full reference for the retraction notice.

*I have performed this check, and no papers have been retracted.*

## Reviewer #1

The paper overall is interesting. The author replies all the questions from previous reviewers.
1. One major concern is that the paper is too long and contains many known works. The author may consider condensing the work and focus more on the major contributions. The author discussed the human brain and neuro; However, it is not clear to see this in the control design part.

*It is good to know that the original submission to PLOS Computational Biology was 11 pages shorter than the current version. The increased length of the current version resulted from taking into account of the criticism of Reviewer #2 of PLOS Computational Biology. Here, I copy his first three points, all of which ask for more information to be included in the paper:*

*“1. The majority of the introduction includes no citations to support the author’s statements. The definition of balance for walking, for example, is well-defined in the literature.*

*2. There has been significant, serious, and quality research on bicycle dynamics in the past 20 years. While the author does identify a subset of the published works, there also exists significant work examining the human control of the bicycle – most work in this area has been led by Dr. Arend Schwab or Dr. Jason Moore. The current study is not a rigorous scientific study, as it largely ignores the existing knowledge and best approaches for evaluating control models.*

*3. The author developed his own model of the bicycle rather than using the validated equations of motion for a bicycle. The bicycle the author uses can easily be obtained using the bicycle model of Meijaard et al. 2007 by simply choosing parameter values. The author spends way too much of the paper talking about the development of his own model, which would be unnecessary if he simply utilized the established linearized equations of motion for a bicycle.”*

2. Is there any experiment validation of the proposed method.

*I did not run an experiment to collect data with which my model for bicycle balance control could be tested. Instead, I used simulations to investigate whether this model is plausible for actual bicycle balance control, and for that I do make use of empirical information. In fact, we know from experience what turn radiuses are physically possible, and what lean angles are acceptable for a rider. Therefore, an empirically plausible model must balance a bicycle without producing turn radiuses that are physically impossible, or lean angles that are unacceptable. My model does this. However, it is extremely sensitive to misestimation of the speed, which is implausible given what we currently about how the CNS estimates speed.*

3. There are still some typos. For instance, in line 26 and line 30, OFC are defined two times.

*Thank you, I corrected those.*

4. It is not common to see “I” in a research paper. Instead, most time we use “We”.

*I am the sole author of this paper. I did a search on the web, I found approximately as many fora/web pages that advise “I” for as single author paper as fora/web pages that advise “We”.*

## Reviewer #2

The work is very interesting and difficult. It should be encouraged. There are some minor problems that I hope can be corrected. When the first time a variable appears, it should be written in its full name. For example, BMX on line 310 and LQR on line 552. In line 1467, the units of curvature should be m^(-1).

*Thank you. I added the full names for BMX and LQR. However, I could not find an error w.r.t. the appropriate units of curvature.*
